# Expression of Concern: Specific Inhibition of Tumor Cells by Oncogenic *EGFR* Specific Silencing by RNA interference

**DOI:** 10.1371/journal.pone.0316041

**Published:** 2024-12-13

**Authors:** The PLOS ONE Editors

After this article [1] was published, concerns were raised about Figs 4 and 5. Specifically, that the right of the PI/TUNEL–siControl panel in Fig 4B appears similar to the left of the PI/TUNEL–Delivery vehicle panel in Fig 5G, despite representing different treatments. During editorial follow-up, the corresponding author acknowledged the panel similarities but stated that the original images underlying Figs 4 and 5 are no longer available. In the absence of the original images, this concern cannot be resolved.

Concerns were also raised that eight pairs of mice shown in Fig 3A and 3D appear similar. The authors stand by the published results and stated that these panels do not include duplicated data. PLOS confirmed that the mouse images in question are not identical, but based on the published images we cannot verify whether they show different animals or different views of the same animals. While this issue has not been fully resolved or dismissed by PLOS, even if considering potential duplications Fig 3A and 3D appear to show at least three different animals per experiment, lending evidence as to the reliability and reproducibility of the results. The corresponding author provided the original images underlying Fig 3A and 3D, which can be viewed here in S1 and S2 Files, however they were unable to provide the associated quantitative data for these figures.

Additionally, concerns were raised regarding vertical discontinuities in both Fig 3A and 3D. The corresponding author stated that each panel is composed of two images due to the limit of three mice per analysis using the IVIS imaging system.

In light of the concerns raised with Figs 4 and 5, the *PLOS ONE* Editors issue this Expression of Concern to notify readers of the above concerns and to relay the available data provided by the corresponding author.

## Supporting information

S1 File Original images underlying Fig 3A including mice from Day 5 and Day 10. (ZIP)

S2 File Original images underlying Fig 3D. (ZIP)
