# Supplementary figures and images for: Expression of Concern: Specific Inhibition of Tumor Cells by Oncogenic EGFR Specific Silencing by RNA interference
Source: PLoS One. 2024 Dec 13;19(12):e0316041. doi: 10.1371/journal.pone.0316041 (PMC11643264; doi:10.1371/journal.pone.0316041)

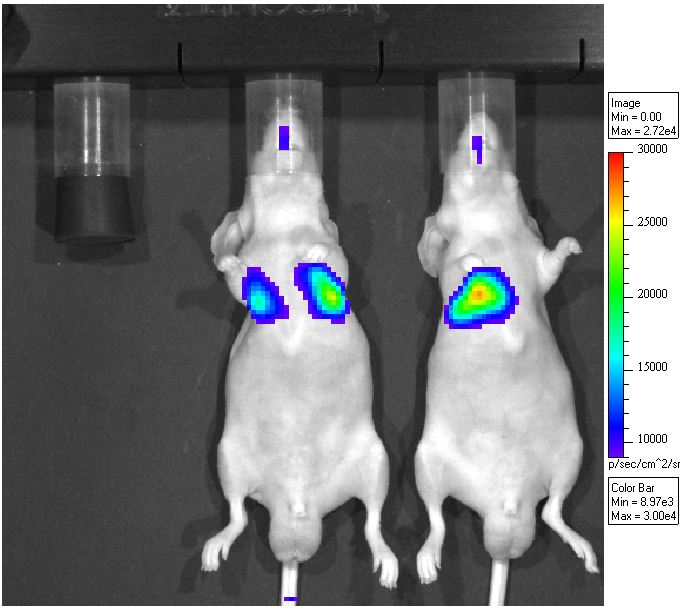

Supplement: S1 File — (ZIP) [file pone.0316041.s001.zip › Day5/siC-2D5.bmp]

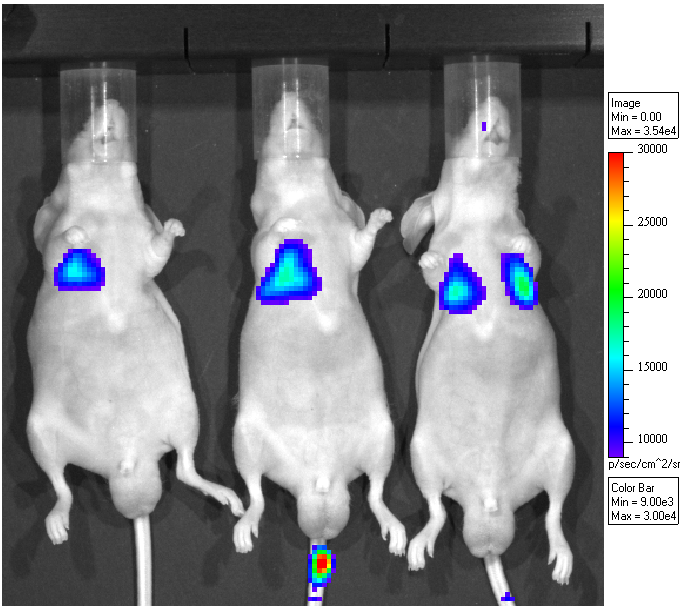

Supplement: S1 File — (ZIP) [file pone.0316041.s001.zip › Day5/siC-3D5.bmp]

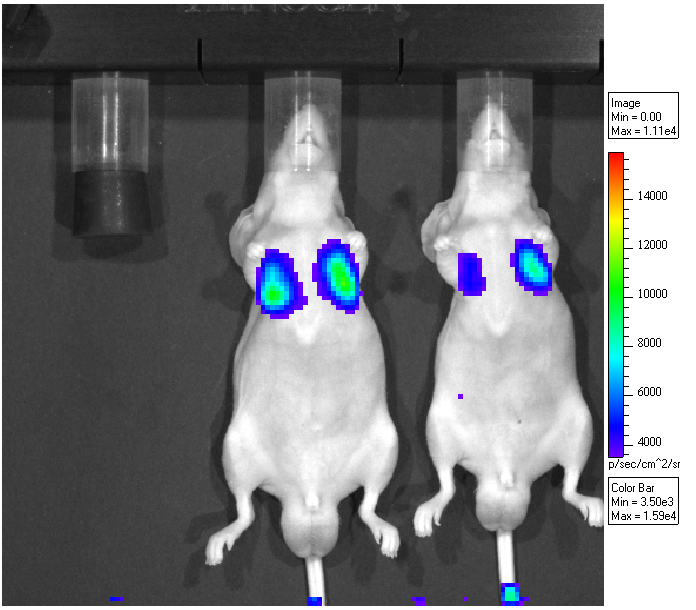

Supplement: S1 File — (ZIP) [file pone.0316041.s001.zip › Day5/siE-2D5.bmp]

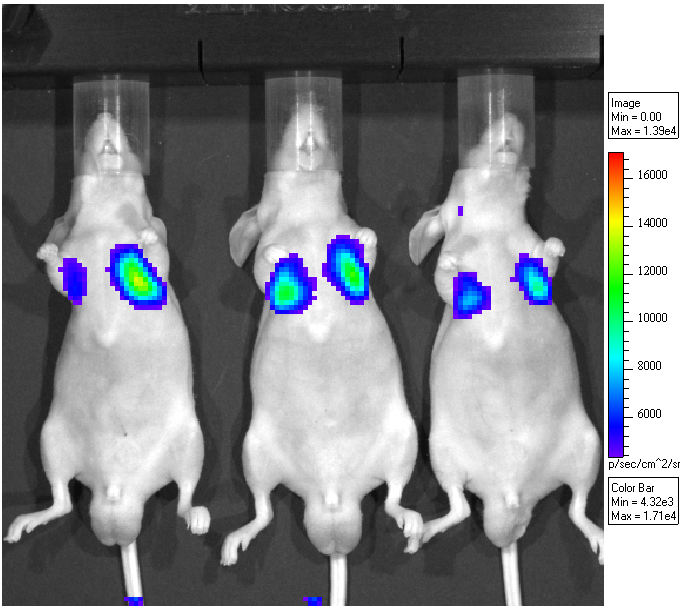

Supplement: S1 File — (ZIP) [file pone.0316041.s001.zip › Day5/siE-3D5.bmp]

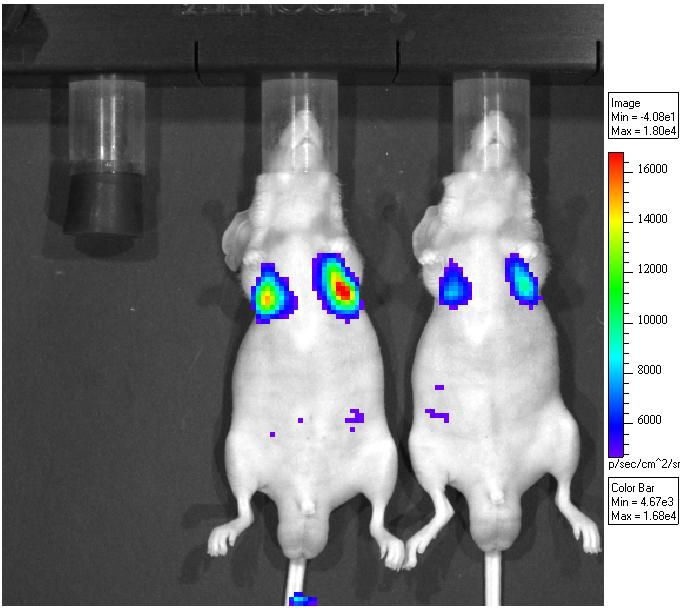

Supplement: S1 File — (ZIP) [file pone.0316041.s001.zip › Day10/siC-2D10.bmp]

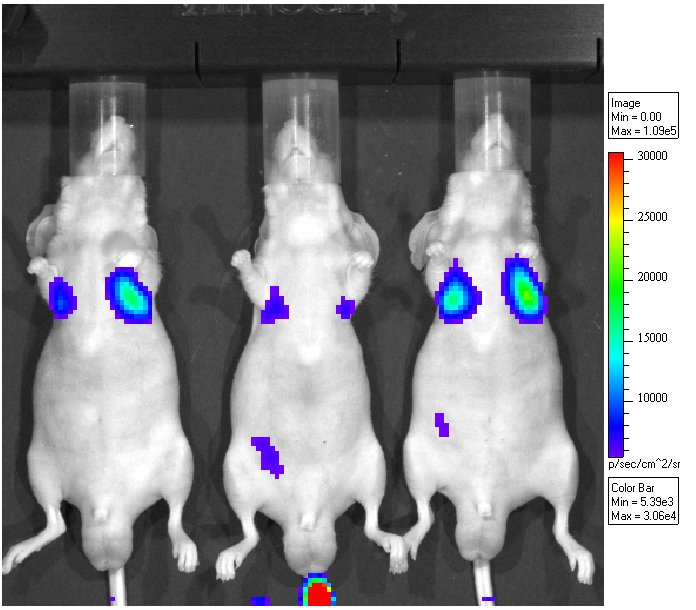

Supplement: S1 File — (ZIP) [file pone.0316041.s001.zip › Day10/siC-3D10.bmp]

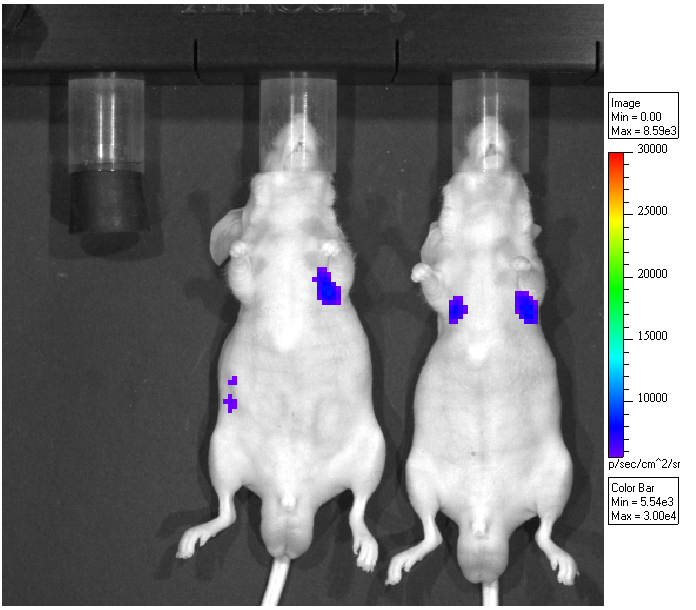

Supplement: S1 File — (ZIP) [file pone.0316041.s001.zip › Day10/siE-2D10.bmp]

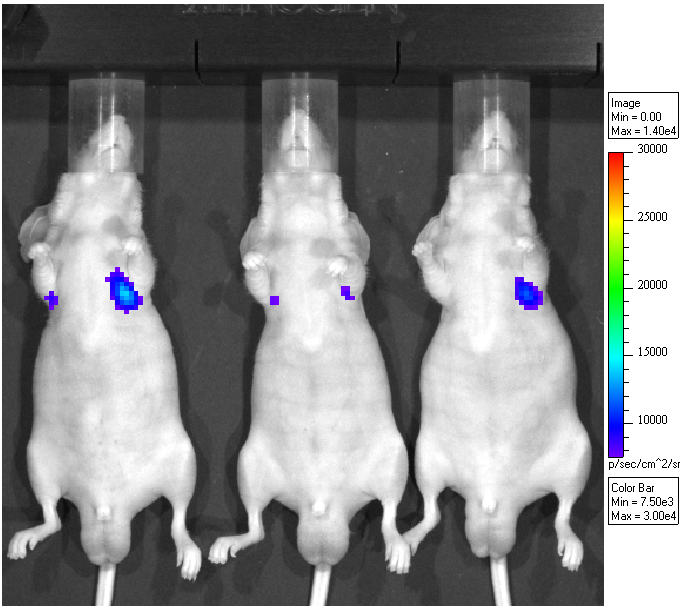

Supplement: S1 File — (ZIP) [file pone.0316041.s001.zip › Day10/siE-3D10.bmp]

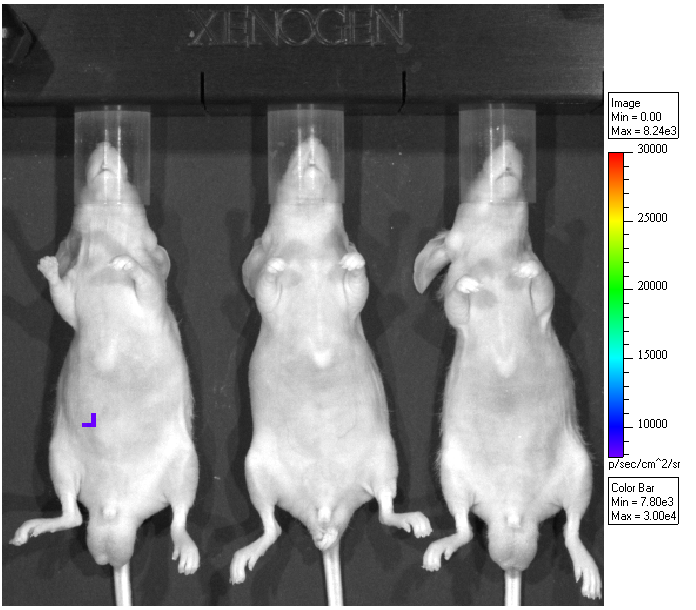

Supplement: S2 File — (ZIP) [file pone.0316041.s002.zip › VivoGlo siC(3-1).bmp]

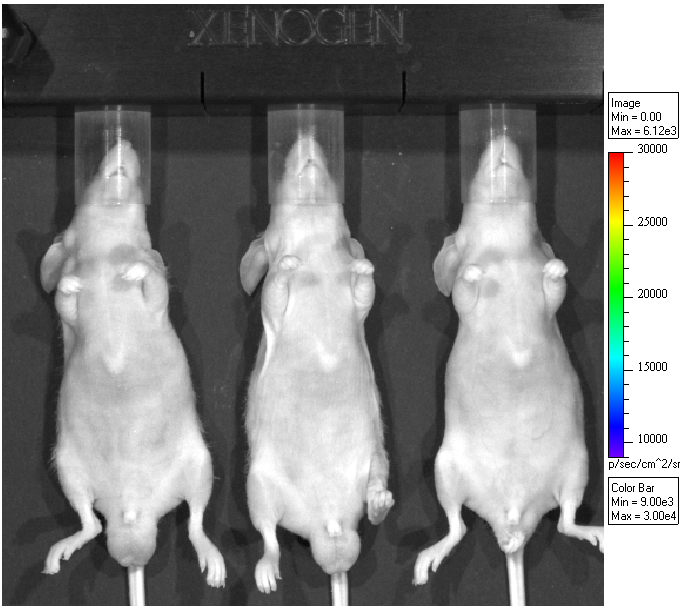

Supplement: S2 File — (ZIP) [file pone.0316041.s002.zip › VivoGlo siC(3-2).bmp]

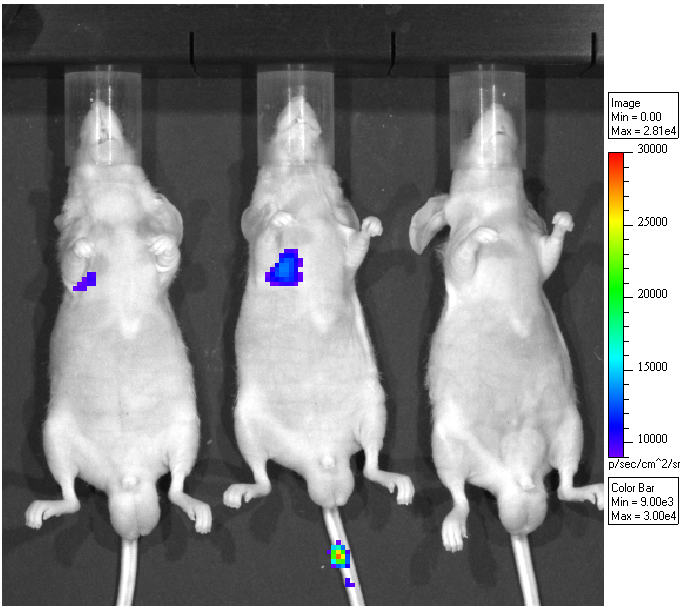

Supplement: S2 File — (ZIP) [file pone.0316041.s002.zip › VivoGlo siE(3-1).bmp]

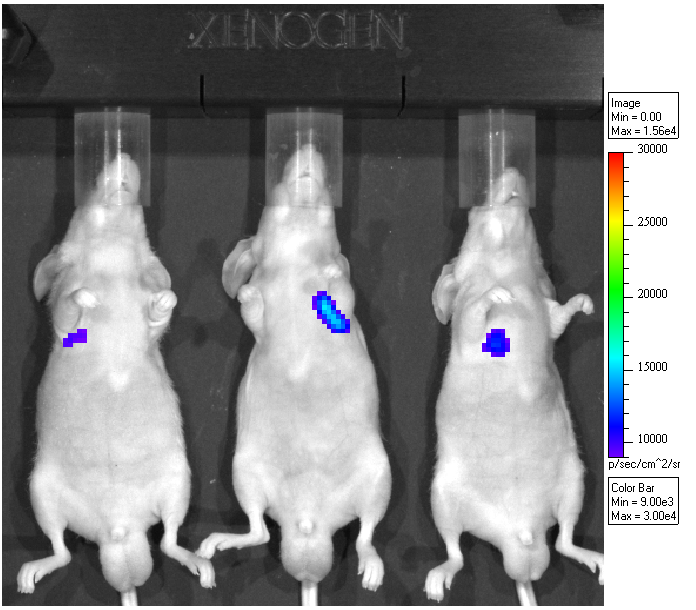

Supplement: S2 File — (ZIP) [file pone.0316041.s002.zip › VivoGlo siE(3-2).bmp]
